# Splitting tensile strength prediction of Metakaolin concrete using machine learning techniques

**DOI:** 10.1038/s41598-023-47196-4

**Published:** 2023-11-16

**Authors:** Qiang Li, Guoqi Ren, Haoran Wang, Qikeng Xu, Jinquan Zhao, Huifen Wang, Yonggang Ding

**Affiliations:** College of Civil Engineering, Henan University of Technology, Zhengzhou, 450001 China

**Keywords:** Energy science and technology, Engineering, Materials science, Mathematics and computing

## Abstract

Splitting tensile strength (STS) is an important mechanical property of concrete. Modeling and predicting the STS of concrete containing Metakaolin is an important method for analyzing the mechanical properties. In this paper, four machine learning models, namely, Artificial Neural Network (ANN), support vector regression (SVR), random forest (RF), and Gradient Boosting Decision Tree (GBDT) were employed to predict the STS. The comprehensive comparison of predictive performance was conducted using evaluation metrics. The results indicate that, compared to other models, the GBDT model exhibits the best test performance with an R^2^ of 0.967, surpassing the values for ANN at 0.949, SVR at 0.963, and RF at 0.947. The other four error metrics are also the smallest among the models, with MSE = 0.041, RMSE = 0.204, MAE = 0.146, and MAPE = 4.856%. This model can serve as a prediction tool for STS in concrete containing Metakaolin, assisting or partially replacing laboratory compression tests, thereby saving costs and time. Moreover, the feature importance of input variables was investigated.

## Introduction

Concrete is the second most consumed material in the world after water. Portland cement is the primary binder used in most concrete applications^1–4^. The production of cement consumes a significant amount of energy and releases approximately 7% of global carbon dioxide emissions into the atmosphere^5–8^. However, the demand for cement continues to rise, and it is projected that annual cement consumption will reach 6 billion metric tons by 2060. One of the methods to reduce cement consumption is to use industrial by-products or more environmentally friendly materials that require less energy during manufacturing, such as Metakaolin. Studies have found that using Metakaolin as a partial replacement for cement can reduce carbon dioxide emissions by up to 170 kg per ton of cement produced^9,10^. Metakaolin is a highly reactive pozzolan that reacts with calcium hydroxide to form C-S–H and aluminate phases^11^. Incorporating Metakaolin as a partial replacement for cement in concrete helps to reduce pore size distribution, improve pore structure, and enhance various mechanical properties ^12–14^.

Previous studies have demonstrated that the addition of Metakaolin can effectively improve the performance of concrete, including enhancing compressive strength and durability, providing better resistance to freezing, weathering, chemical erosion, and permeability, as well as improving early-age concrete properties^15,16^. Therefore, estimating the mechanical properties of concrete, such as splitting tensile strength (STS), based on concrete mix proportions can help save time and costs, facilitate activities such as formwork removal, and promote the application of Metakaolin in the concrete industry. Previous research has mainly focused on finding the optimal Metakaolin content required to ensure the desired mechanical properties of concrete through experimental approaches^17,18^. However, the experimental process is time-consuming and labor-intensive. It would be highly useful to establish an intelligent model based on previous experimental data to predict the mechanical properties under given input mix proportions, which will significantly save experimental time and testing costs.

With the development of artificial intelligence, an increasing number of algorithms and models have provided new perspectives for addressing these problems^19–34^. The implementation of intelligent models for predicting the mechanical properties of concrete has also gained increasing attention^35–46^. Wu et al. ^47^ achieved accurate prediction of high-performance concrete tensile strength by utilizing a combination of support vector regression (SVR) and artificial neural network (ANN) models with optimization algorithms. Sourav et al. ^48^ employed support vector machine (SVM) and gradient boosting machine (GBM) models to predict the tensile strength of concrete, and the results indicated that GBM outperformed SVM in terms of prediction performance. Hammad et al. ^49^ utilized four models, namely gene expression programming (GEP), ANN, M5P model tree algorithm, and random forest (RF), to predict the flexural strengths of concrete with metakaolin, and the results demonstrated that random forest achieved the best predictive performance. Nozar et al. ^50^ studied the compressive strength of concrete containing metakaolin using the Multi-Layer Perceptron (MLP) model, and the results showed that the MLP network had reliable accuracy in predicting the compressive strength of concrete with metakaolin. Furthermore, user-friendly software was developed to facilitate the use of the proposed MLP network based on machine learning methods. Huang et al. ^51^ proposed a hybrid machine learning model combining RF and firefly algorithm (FA) to accurately predict the compressive strength of cementitious materials containing expansive clays based on a database of 361 samples. Abdulrahman et al. ^16^ compared the predictive performance of multiple individual models and ensemble models in predicting the compressive strength of cementitious materials containing expansive clays, and it was found that the DT AdaBoost model and the improved bagging model achieved the best predictive performance in predicting the STS of Metakaolin concrete.

**Figure 1:**
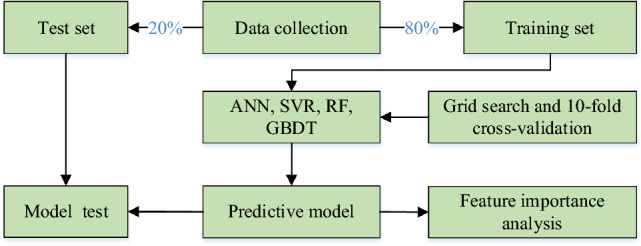
Paper framework workflow.

However, there is relatively limited research and analysis on using machine learning models to predict the STS of concrete containing Metakaolin. Further research is needed in this area. Therefore, this paper aims to model and compare the STS of concrete containing Metakaolin using individual and ensemble models based on variables such as cement, Metakaolin, water-to-binder ratio (w/b), fine aggregate (FA), coarse aggregate (CA), superplasticizer (SP), age, height (H), and diameter (D) of concrete column specimen. The framework of this study is illustrated in Fig. 1.

## Machine learning models

### Artificial neural network

**Figure 2:**
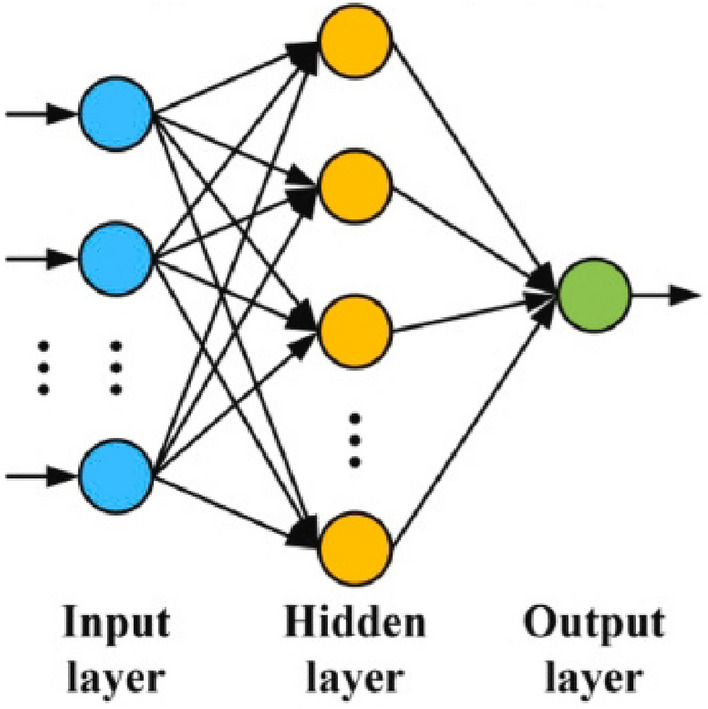
ANN network structure.

ANN is a useful machine learning technique based on biological neural networks, designed to simulate complex relationships between inputs and outputs. The simplest processing element in a neural network is a neuron. Each neuron *i* may have multiple inputs, *x*_*1*_, *x*_*2*_, …, *x*_*d*_, which are combined with corresponding weights, *w*_*i1*_, *w*_*i2*_, …, to produce a single output. More specifically, the propagation function combines these inputs with their weights and then applies an activation function to the resulting sum to generate the corresponding output ^52^. The structure of an ANN is depicted in Fig. 2.

### Support vector regression

**Figure 3:**
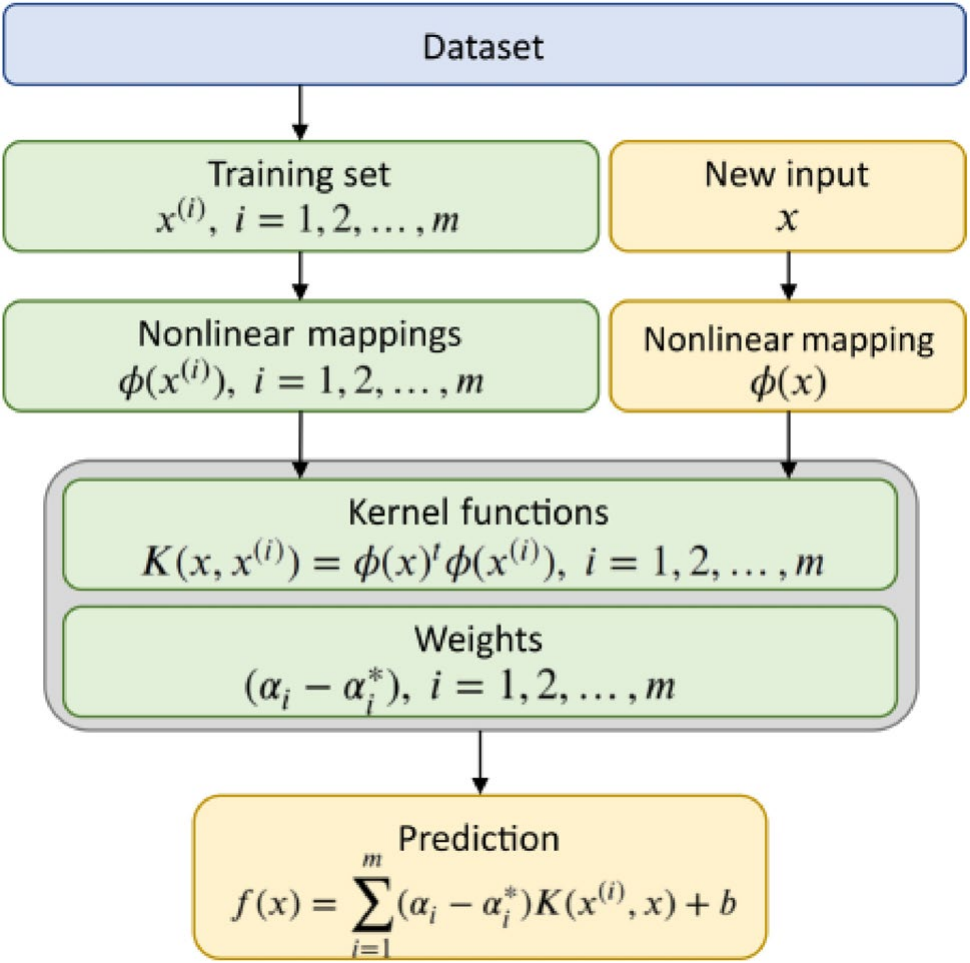
SVR algorithm flow^54^.

SVM was originally proposed for studying linear problems. The basic idea behind SVM for pattern recognition is to transform the input space into a high-dimensional space through a non-linear transformation^53^. In this new space, the algorithm solves a convex quadratic programming problem to find the optimal linear classification hyperplane. However, when used for regression prediction, the fundamental idea is not to find an optimal classification plane that separates the samples but to find an optimal hyperplane that minimizes the distance between the hyperplane and all training samples. This hyperplane can be considered a well-fitted curve, and the approach of using SVM for function approximation is known as SVR. SVR can be summarized as using a non-linear mapping function to map the input samples to a high-dimensional feature space and learning a linear regression quantity in the feature space to obtain the estimation function. The steps for implementing SVR for regression prediction are illustrated in Fig. 3.

### Random forest

**Figure 4:**
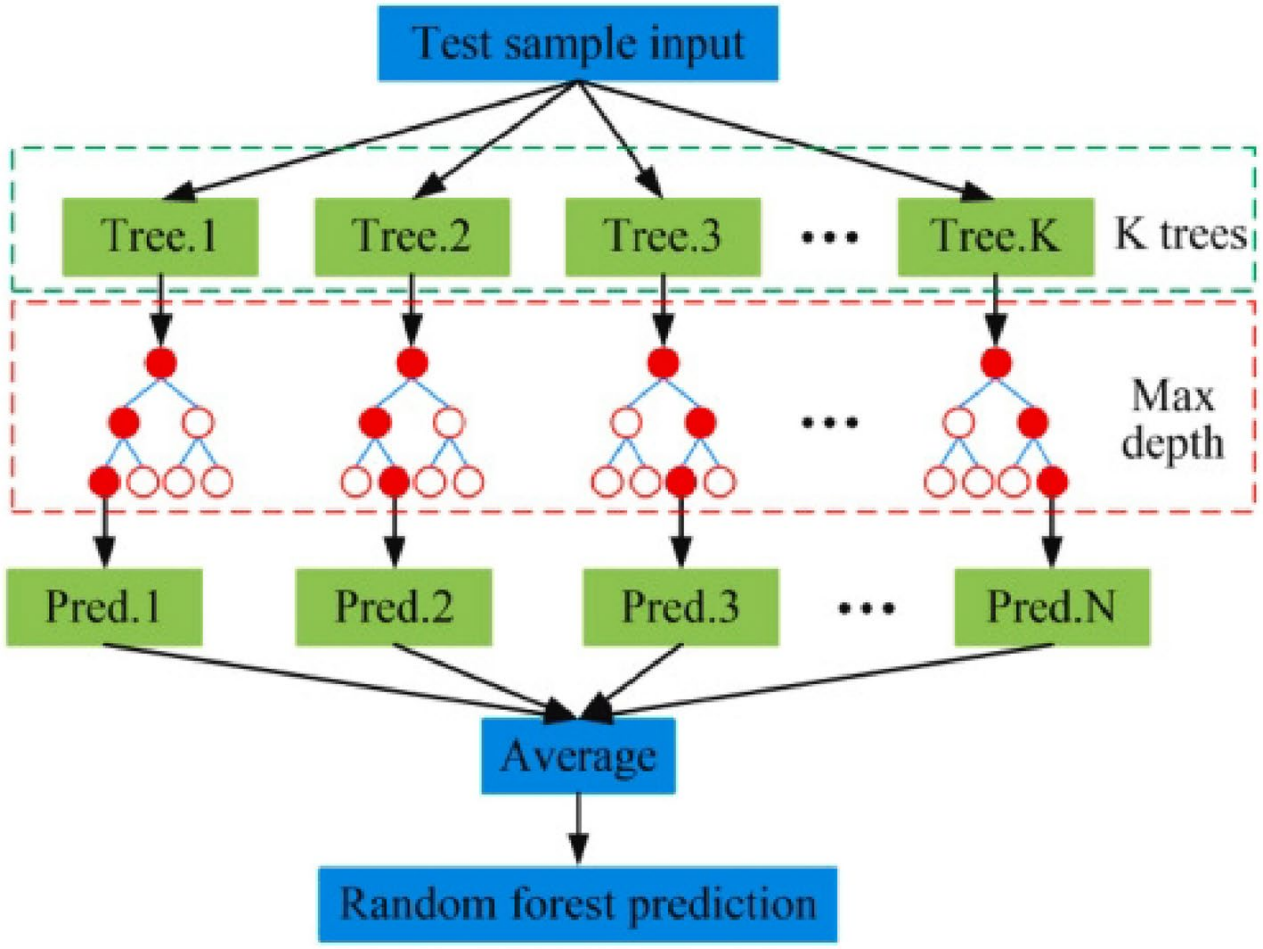
Flowchart of random forest model^55^.

RF is an integrated learning model consisting of multiple decision trees. Its core idea is to improve prediction accuracy and stability by constructing multiple decision trees. As shown in Fig. 4, each decision tree is constructed based on random samples and random features, and this randomness makes Random Forest able to avoid overfitting and has good robustness. Advantages include: (1) Since random forests can utilize multiple decision trees for prediction, their prediction accuracy is higher than that of a single decision tree. (2) Random forests can handle a large number of input features, so they can be used for classification and regression problems with high-dimensional data. (3) Random forests are constructed using random samples and random features, and this randomness avoids the problem of overfitting.

### Gradient boosting decision tree

Gradient Boosting Decision Trees (GBDT) is established based on the Boosting method in ensemble learning. It requires multiple iterations and the construction of multiple decision trees to form an ensemble model. During each iteration, the decision tree learners reduce the residuals along the direction of the steepest gradient descent. The algorithm is widely applied due to its strong interpretability, fast prediction speed, and the ability to freely combine multiple influential factors. When constructing the model, there is a strong correlation between each decision tree. Each subsequent decision tree adjusts its own weights based on the training results of the previous decision tree, and this process iterates until the desired residual or the maximum number of iterations is reached. The predictive model of GBDT can be represented as:

1 $$F(x) = \sum\limits_{k = 1}^{K} {\omega_{k} g(x,\varphi_{k} )}$$

where $F(x)$ is the response value of the input variable *x*; $\omega_{k}$ and $\varphi_{k}$ are the weights and parameters of the *k*-th decision tree, respectively; and $g(x,\varphi_{k} )$ is the predicted value of the *k*-th decision tree.

## Dataset collection

**Figure 5:**
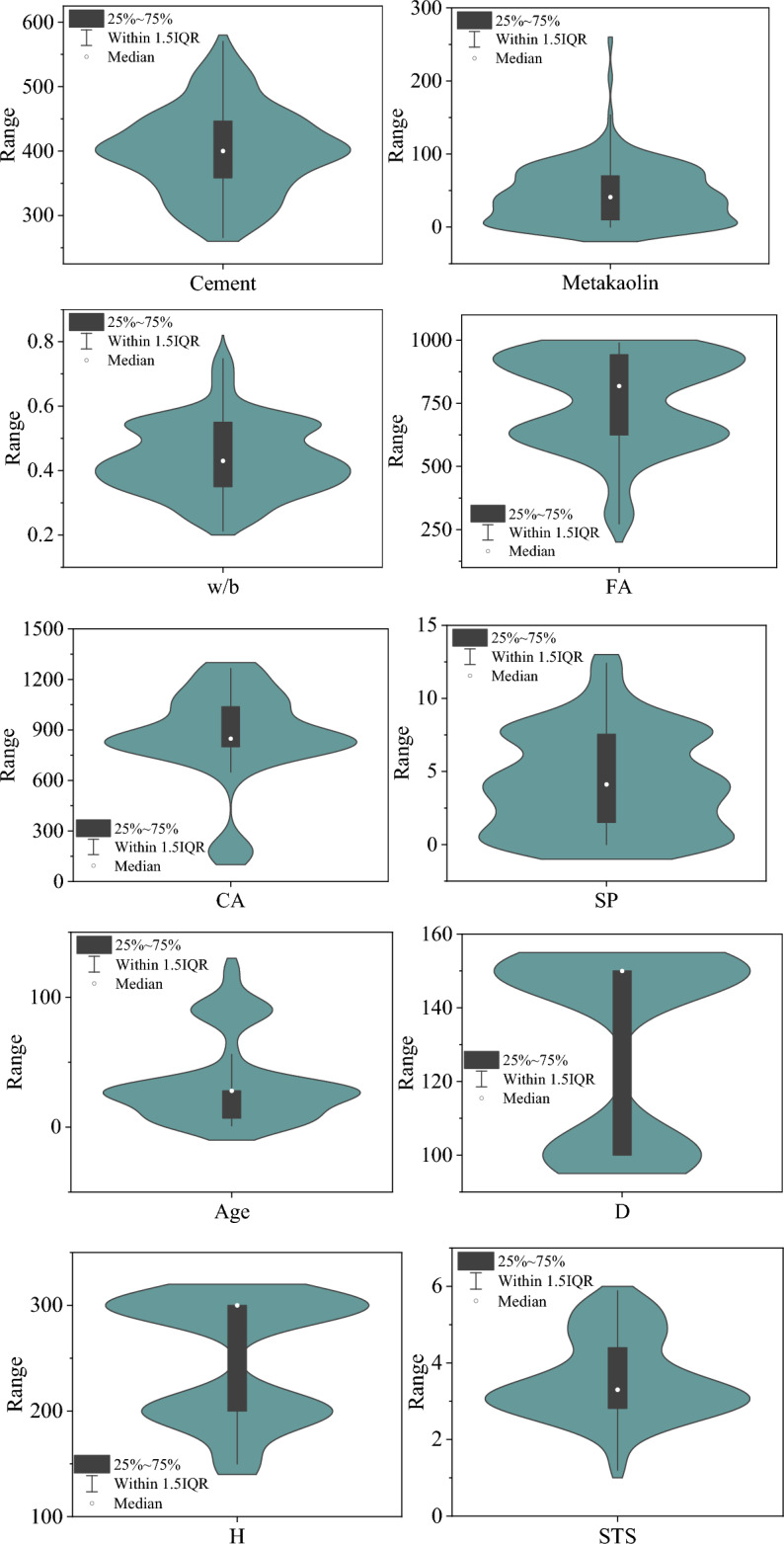
Distribution histogram of variables.

**Figure 6:**
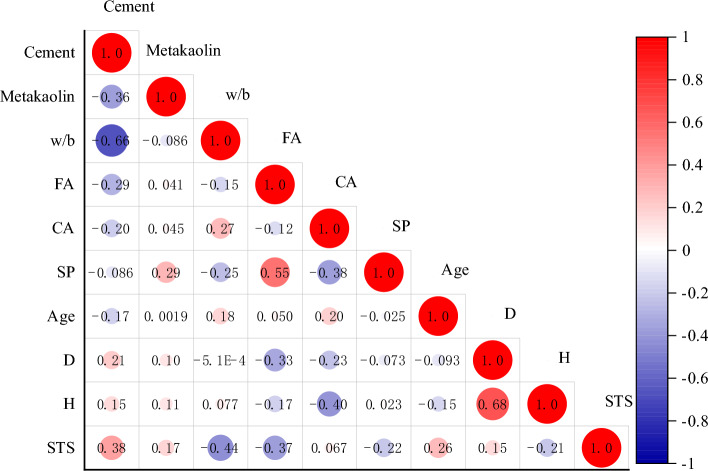
Pearson correlation coefficients between variables.

For machine learning models, a representative dataset is necessary and important. Therefore, this study collected a total of 204 samples from the literature^17,18,56–70^. The descriptive statistics and histogram distributions of the variables in these samples are shown in Table 1 and Fig. 5, respectively, where the input variables include component ratio, curing age, and specimen size. It can be observed that the content of "Metakaolin" ranges from 0 to 256 across the entire dataset, indicating a high degree of data variability. Furthermore, Fig. 6 presents the Pearson correlation coefficients between the variables. It can be seen that among the 9 input features listed, the linear correlation between "cement", "w/b" and "STS" is the strongest, with correlation coefficients of 0.3776 and −0.4362 respectively. However, this correlation is still weak, indicating that relying on multiple linear regression for predicting STS is unreliable due to the existence of complex nonlinear relationships between these variables and the output. This is why this study adopts machine learning models to achieve accurate predictions of STS. Moreover, the linear correlation between the input variables is weak, which is also an important prerequisite for machine learning applications.

**Table 1:** Characteristics of the variables.

Variable	Cement	Metakaolin	w/b	FA	CA	SP	Age	D	H	STS
Type	Input	Input	Input	Input	Input	Input	Input	Input	Input	Output
Size	204	204	204	204	204	204	204	204	204	204
Unit	kg/m^3^	kg/m^3^	/	kg/m^3^	kg/m^3^	kg/m^3^	Day	mm	mm	MPa
Max	570.00	256.00	0.75	989.00	1264.97	12.40	120.00	150.00	300.00	5.88
medium	400.00	41.00	0.43	818.10	848.00	4.12	28.00	150.00	300.00	3.30
Min	266.00	0.00	0.21	272.50	175.10	0.00	1.00	100.00	150.00	1.20
Std	65.63	38.86	0.11	180.72	266.44	3.34	31.66	24.30	56.21	1.05
Average	400.42	43.83	0.44	756.64	865.01	4.23	34.66	130.88	248.53	3.58
Cov	0.16	0.89	0.25	0.24	0.31	0.79	0.91	0.19	0.23	0.29
Skewness	0.13	1.33	0.40	− 0.58	− 1.11	0.41	1.22	− 0.49	− 0.32	0.44
Kurtosis	− 0.36	4.29	− 0.02	− 0.39	1.60	− 0.69	0.35	− 1.78	− 1.59	− 0.51

## Results and analysis

### Model building

A total of 163 samples (80%) were randomly selected as the training set, and the remaining 41 samples (20%) were used as the test set for the trained model. After splitting the data, normalize the features to [0,1] to avoid scale effects. Referring to the literature^71^, tenfold cross-validation and grid search methods were used to obtain the optimal hyper-parameters. The parameter value was determined in Table 2.

**Table 2:** Parameters setting for different models.

Model	Parameters	Value
ANN	Hidden layer	2
	Hidden neuron	55
SVR	kernel	RBF
	C	3
	gamma	0.25
RF	Estimators	48
	Maximum depth of the tree	10
	Minimum samples for split	2
	Minimum samples of leaf node	1
GBDT	Estimators	80
	Learning rate	0.25
	Minimum samples for split	6
	Maximum depth of the tree	3
	Minimum samples of leaf node	5

### Performance comparison

**Figure 7:**
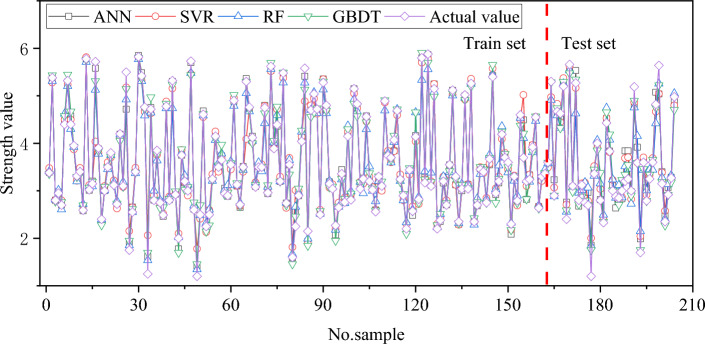
Comparison between actual and predicted values of each sample for different models.

**Figure 8:**
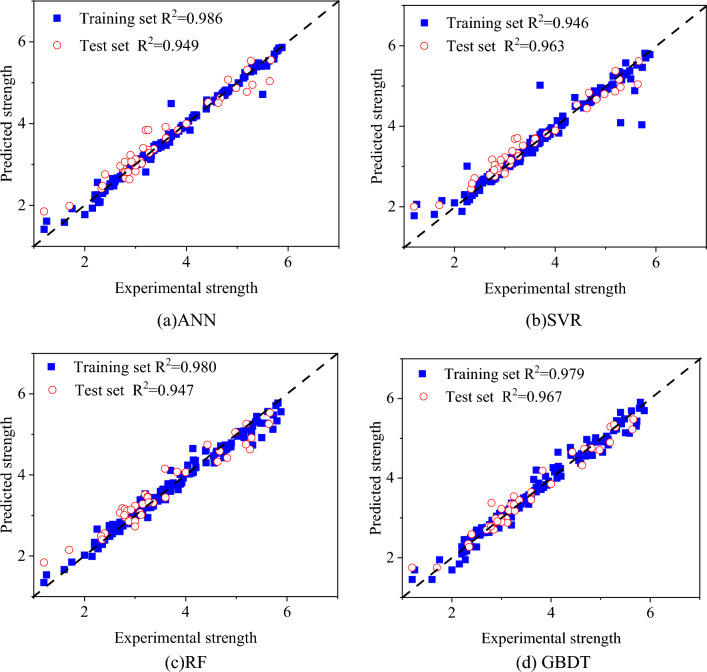
Correlation between predicted and actual values for different models.

Figure 7 illustrates the deviations between the predicted results and the actual results of each sample for different models. The training and testing results of different models are shown in Fig. 8. From the perspective of the coefficient of determination (R^2^), all four models achieve good predictive performance. Among them, the GBDT model achieved the highest correlation coefficient of 0.967, followed by 0.963 for SVR, 0.949 for ANN, and 0.947 for RF. In general, relying on a single metric for evaluation may be unreliable. Therefore, four error metrics for each model's predictions were calculated, as shown in Table 3. It can be observed that compared to the other three models, the GBDT model achieves smaller error metrics. Specifically, the MSE, RMSE, MAE, and MAPE for the GBDT model are 0.041, 0.204, 0.146, and 4.856%, respectively.

**Table 3:** Evaluation index calculation of different model test results.

Evaluation index	ANN	SVR	RF	GBDT
R^2^	0.949	0.963	0.947	0.967
MSE	0.071	0.066	0.083	0.041
RMSE	0.266	0.256	0.287	0.204
MAE	0.196	0.194	0.236	0.146
MAPE/%	6.716	6.948	7.841	4.856

**Figure 9:**
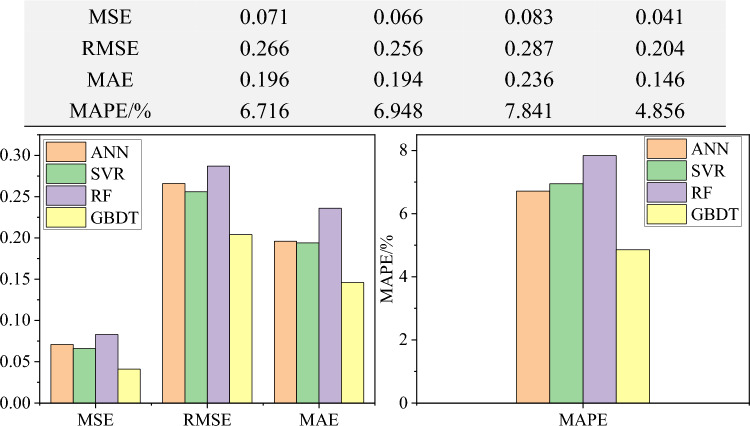
Histogram of evaluation indicator for test set.

**Figure 10:**
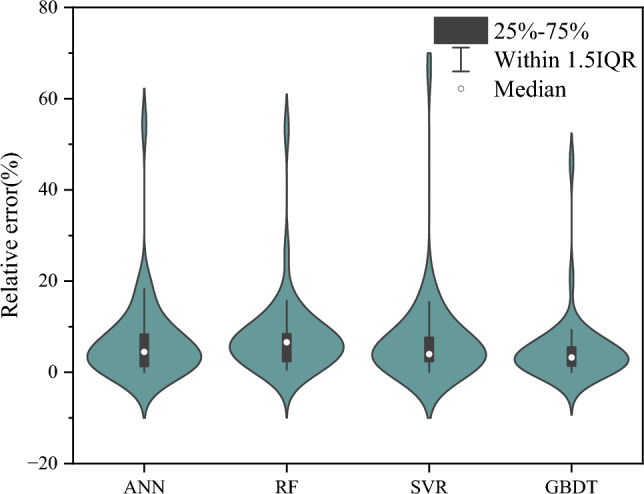
The violin diagram for relative error percentage of different models.

For a more intuitive comparison, Fig. 9 presents the histograms of different model evaluation metrics. It can be concluded that overall, the GBDT model exhibits the best predictive performance among the machine learning models. Figure 10 shows a violin plot of the relative error percentages for different models. It can be observed that, compared to other models, the GBDT model exhibits a more concentrated and closer-to-zero relative prediction error in the test dataset. The statistical analysis of the errors further underscores the positive predictive performance.

### Feature importance

**Figure 11:**
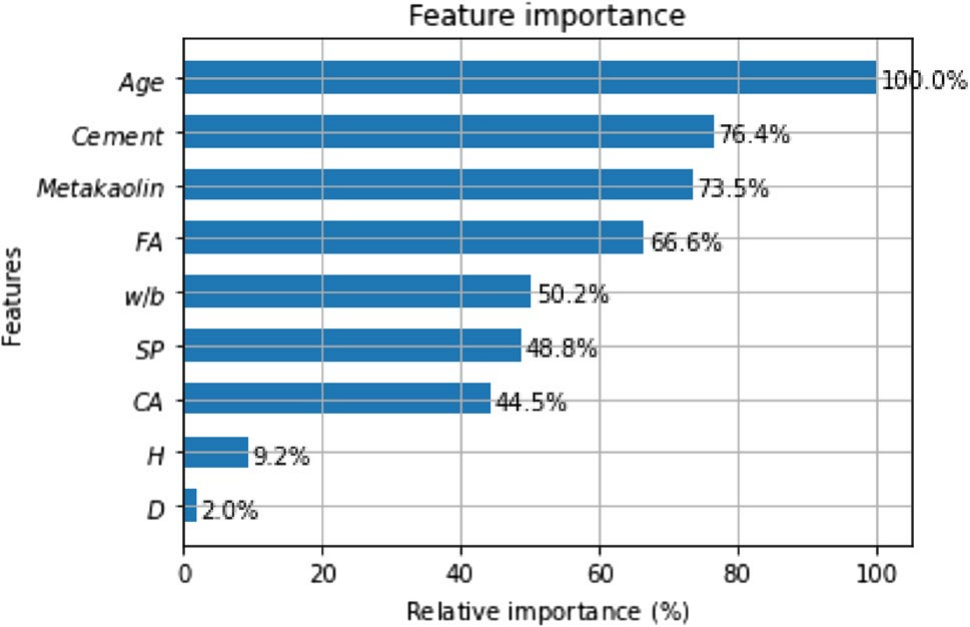
Feature importance analysis.

Feature importance analysis is the most commonly used method for interpreting model outputs. This analysis directly indicates the degree of influence of each feature on the final predictions. The greater the impact of a feature on the model's predictions, the more significant it is. Figure 11 presents the relative importance results of various features in predicting STS output using the GBDT model. Age is the most important feature for STS, which is as expected, as different ages exhibit significant differences in mechanical performance. Normalizing the relative importance of Age to 100%, the subsequent importance rankings are Cement and Metakaolin, with their importance being approximately three-quarters of the Age. Following that, in descending order, are FA, w/b, SP, and CA. The dimensions of the specimens are less important features, accounting for 9.2% for H and 2% for D.

## Conclusions

This study proposes an STS prediction method involving concrete containing Metakaolin using individual and ensemble learning models. These machine learning models demonstrate good performance in reflecting the complex nonlinear relationships between input and output parameters in the prediction of STS for concrete containing Metakaolin. Based on the correlation coefficient between the predicted results and actual values, and considering other error metrics, the GBDT ensemble model exhibits the best prediction performance and is recommended as an intelligent method for STS prediction.

In the current dataset, the feature importance analysis based on the GBDT model shows that the most influential feature affecting STS is Age, followed by Cement, Metakaolin, FA, w/b, SP, and CA. The specimen dimensions have a relatively minor impact on STS. Feature importance analysis can provide guidance for obtaining the expected STS of Metakaolin concrete.

Although the machine learning methods developed in this study have achieved good prediction results, it should be noted that the research is conducted on a specific dataset. In the future, it is necessary to expand the dataset with more samples and search for samples that encompass a wider range of input parameters. Moreover, using Shapley Additive explanations analysis to further investigate the impact of these features on the output is also a focal point of future research.

## Data Availability

The datasets used during the current study available from the corresponding author on reasonable request.
